# Bifunctional Single-Atom Cobalt Electrocatalysts with Dense Active Sites Prepared via a Silica Xerogel Strategy for Rechargeable Zinc–Air Batteries

**DOI:** 10.3390/nano12030381

**Published:** 2022-01-24

**Authors:** Lijuan Wang, Zixiang Xu, Tingyu Peng, Maosong Liu, Long Zhang, Jianming Zhang

**Affiliations:** 1 Institute of Quantum and Sustainable Technology (IQST), School of Chemistry and Chemical Engineering, Jiangsu University, Zhenjiang 212013, China; 2211912020@stmail.ujs.edu.cn (L.W.); 3191303039@stmail.ujs.edu.cn (Z.X.); 2 Instrumental Analysis Center, Jiangsu University, Zhenjiang 212013, China; 1000005654@ujs.edu.cn

**Keywords:** single-atom catalysts, ORR/OER, electrocatalysis, zinc–air battery, xerogel

## Abstract

The N-doped cobalt-based (Co) bifunctional single atom catalyst (SAC) has emerged as one of the most promising candidates to substitute noble metal-based catalysts for highly efficient bifunctionality. Herein, a facile silica xerogel strategy is elaborately designed to synthesize uniformly dispersed and dense Co-N_x_ active sites on N-doped highly porous carbon networks (Co-N-C SAC) using economic biomass materials. This strategy promotes the generation of massive mesopores and micropores for substantially improving the formation of Co-N_x_ moieties and unique network architecture. The Co-N-C SAC electrocatalysts exhibit an excellent bifunctional activity with a potential gap (ΔE) of 0.81 V in alkaline medias, outperforming those of the most highly active bifunctional electrocatalysts. On top of that, Co-N-C SAC also possesses outstanding performance in ZABs with superior power density/specific capacity. This proposed synthetic method will provide a new inspiration for fabricating various high-content SACs for varied applications.

## 1. Introduction

The metal–air battery has attracted considerable attention as a promising energy storage system due to its high theoretical energy/power density, reliable safety, and economic viability [1,2,3,4]. Among them, alkaline Zn–air batteries (ZABs) have been widely studied owing to the advantages of being cost-effective and their high theorical energy density up to 1084 W h kg^−1^ [5,6,7]. However, the main impediment of their commercial application is the sluggish kinetics and poor durability of the oxygen evolution reaction (OER) and oxygen reduction reaction (ORR) on the air electrode [8,9,10,11,12]. Generally, the two reactions still need noble metal catalysts (such as Ir/Ru for OER, Pt for ORR), which suffer from the severely unavoidable deficiencies such as high cost, scarcity, and poor long-term stability [13,14,15]. Thus, marvelous efforts have been devoted to exploring cost-effective materials as the ORR and OER bifunctional catalyst with high activity to substitute precious Ru-, Ir-, and Pt-based catalyst, such as metal oxides, sulfides, hydroxides, single atom catalysts (SACs), and other hybrids [16,17,18,19,20,21,22]. Among all the previously reported non-precious metal catalysts, heteroatom-doped bifunctional SACs are attracting ever-growing interest for ZABs. For example, single atomic cobalt coordinated nitrogen (Co-N_x_) moieties in the carbon matrix have been selected as promising low-cost substitutes for ZABs, contributing their elemental abundance, high atom utilization, and impressive bifunctional activity [5,6,7,23,24].

A series of strategies based on co-precipitation, wet impregnation, and metal–organic frameworks (MOFs) [25,26,27] have been recently developed for preparing Co-N-C SACs. Furthermore, many advanced biomass-derived electrocatalysts, e.g., nutshell and red bean pod, have shown excellent electrocatalytic performance for metal–air batteries [28,29,30]. Despite significant progresses, there are still quite a few challenges to synthesize the well-defined metal–nitrogen co-doped carbon catalysts, including the aggregation of metal atoms at high temperature, harsh reaction conditions, and environmental pollutions. These side effects greatly limit exact investigations of the catalytic mechanism and activity [31,32]. In order to produce a high content of Co-N_x_ moieties in Co-N-C SACs for efficient OERs and ORRs, the pyrolysis of concentrated Co- and N-containing precursors with C source is the most prevailing manipulation for generating adequate active sites; however, the Co atoms inevitably tend to agglomerate and cluster into Co-based crystals or nanoparticles encapsulated by C, driven by their high surface free energy during thermal treatment. This phenomenon will be further intensified with the concentration increase in Co precursor, thereby seriously suppressing the formation of active Co-N_x_ sites as well as inducing decreased Co-loading in SACs (<1 wt%) [6,33,34,35,36,37,38,39,40,41]. Apart from the stabilization of Co, the porosity of the carbon matrix is another key factor for the OER and ORR activity, which exerts an important impact on hosting active sites and the accessibility of reactants to the three-phase boundary. In particular, micropores mainly host the Co-N_x_ active sites, and mesopores can facilitate the mass transport of reactants [42,43,44]. Thus, it is necessary to select a suitable substrate to assist the synthesis of densely well-defined SACs with optimized pore distribution.

Herein, we report a silica xerogel-assisted synthetic approach to fabricate atomically dispersed Co on a N-doped porous network C (shortened as Co-N-C SAC thereafter) using the Stöber method and naturally abundant materials of cobalt gluconate and glucosamine as the Co, N, and C precursors. Benefiting from the dense Co-N_x_ moieties and rational porous microstructure of the resultant C materials, the as-prepared Co-N-C SAC demonstrates excellent bifunctional OER/ORR activity with an overpotential ΔE of 0.81 V in alkaline medium, which is better than most of the bifunctional oxygen catalysts reported previously [45,46]. The assembled ZAB using the Co-N-C SACs shows a high open-circuit voltage of 1.49 V, a high-power density of 143.1 mW cm^−2^, and high specific capacity (942 mA h g^−1^) at 10 mA cm^−2^, surpassing the commercial 20% Pt/C + IrO_2_ catalyst and most of the non-precious-metal catalysts [47,48,49]. This facile strategy, based on biomass-derived precursors, can open an avenue for the large-scale commercial production of single-atom catalysts.

## 2. Experimental Section

### 2.1. Materials

Sodium gluconate, glucosamine hydrochloride, cobalt chloride hexahydrate (CoCl_2_·6H_2_O), tetraethyl orthosilicate (TEOS), ammonia (25–28 wt%), and absolute ethanol were all analytical grade, commercially available from Shanghai Chemical Reagent Co., Ltd. (Shanghai, China), and used without further purification. The commercial Pt/C (20 wt%) and IrO_2_ catalysts were purchased from Johnson Matthey Corporation (London, UK). Deionized water (18 MΩ·cm) was used in the whole experiments.

### 2.2. Synthesis of Co-N-C SAC Catalyst

Glucosamine hydrochloride (1.5 g), sodium gluconate (0.3 g), and CoCl_2_·6H_2_O (0.15 g) were first dissolved in 13 mL of H_2_O containing 50 μL of ammonia. Then, absolute ethanol (10 mL) and TEOS (10 mL) were introduced to the above transparent mixture and kept for 3 h at 60 °C under stirring to form the glucose/SiO_2_ sol–gel. Glucose/SiO_2_ xerogels were obtained by freeze drying the resulting sol–gel for 12 h. The dry powder was subsequently ground with the same quantity of urea, which was followed by transferring the crude into a furnace and pyrolyzing at 900 °C for 2 h under the argon (Ar) atmosphere. The pyrolyzed sample (denoted as Co-N-C/SiO_2_) was etched using a hydrofluoric acid solution to remove silica and Co particles. The acid-washed sample was thermal-treated at 900 °C for another 2 h in Ar to form the final product of Co-N-C SAC. Using the same protocol as the preparation of Co-N-C SAC, the N-C control catalyst was prepared in the absence of CoCl_2_·6H_2_O.

## 3. Characterizations

Transmission electron microscopy (TEM) and X-ray photoelectron spectroscopic (XPS) measurements were performed using Talos-200X with EDS analysis and ESCALAB Xi+, respectively (Thermo Fisher Scientific, Waltham, MA, USA). Scanning electron microscopy (SEM) was tested using a JSM-7800 (JEOL Co., Ltd., Tokyo, Japan). XPS peak energies were calibrated by placing the graphite C 1s peak at 284.6 eV. The aberration-corrected high-angle annular dark-field scanning TEM (HAADF-STEM) was conducted using a Spectra 300 at the Center for Electron Microscopy of Thermo Fisher Scientific, Eindhoven, Netherlands. X-ray diffraction (XRD) was investigated using a Rotaflex D/MAX-2500 (Rigaku Co., Ltd., Tokyo, Japan), with Cu Kα radiation (λ = 1.54178 Å). BET experiments were conducted at 77 K on a Quantachrome AUTOSORB IQ Instrument (Anton Paar Quanta Tec Inc., Boynton Beach, FL, USA). The surface areas were estimated from the Brunauer–Emmett–Teller (BET) equation by fitting the N_2_-adsorption isotherms from 0.05 to 0.3 (P/P_0_ range). Raman spectra were recorded using a Thermo Fisher DXR (Thermo Fisher Scientific) with an incident laser wavelength of 532 nm.

### 3.1. Electrochemical Measurements

The electrochemical measurements were conducted using a CHI 760D potentiostat with a one-component three-electrode cell in 0.1 M KOH electrolyte. A platinum wire and an Ag/AgCl electrode were used as the counter and reference electrode, respectively. The working electrode was a glassy-carbon (GC) rotating ring disk electrode (RRDE) (diameter: 5 mm, area: 0.196 cm^2^) purchased from Pine Instruments (Grove City, PA, USA). The measured potential against Ag/AgCl was converted to an RHE value using the Nernst equation: E_RHE_ = E_Ag/AgCl_ + 0.059 pH + 0.197. All the measurements were measured at ambient conditions. The catalyst ink was prepared by dispersing 5 mg of the catalyst into 480 μL ethanol containing 40 μL of Nafion (5%). Then, the catalyst ink was deposited on the GC electrode with an overall catalyst loading of 0.3 mg cm^−2^. The cyclic voltammetry (CV) curves were recorded in both N_2_- and O_2_-saturated electrolytes with a 50 mV s^−1^ of scan rate. In contrast, commercial 20 wt% Pt/C and IrO_2_ catalyst (Johnson Matthey, JM) was used as the control catalyst, and the Pt loading was 0.1 mg cm^−2^. The ORR and OER activity were determined by linear scanning voltammetry (LSV) on an RRDE in an O_2_-saturated and N_2_-saturated 0.1 M KOH solution with a rotating speed of 1600 rpm, respectively. All LSV measurements of the catalysts were measured using an RRDE.

The Koutecky–Levich (*K*–*L*) plots can be obtained by linear fitting of the reciprocal rotating speed versus reciprocal current density collected at −0.2 V, −0.3 V, −0.4 V, −0.5 V, and −0.6 V, respectively. The electron transfer number (*n*) of the sample can be calculated by *K*–*L* plots according to the ORR-LSV data at different rotation rates (Equations (1) and (2)).

(1) $$\frac{1}{J}=\frac{1}{J_{K}}+\frac{I}{J_{L}}=\frac{1}{J_{K}}+\frac{1}{B}\omega^{-\frac{1}{2}}$$

(2) $$B=0.2nF{\left(D_{\left(O_{2}\right)}\right)}^{\frac{2}{3}}\nu^{-\frac{1}{6}}C_{\left(O_{2}\right)}$$

where *J* and *J_K_* are the measured current density and kinetic current density, respectively, *F* is the Faraday constant (96485 C mol^−1^), $D_{\left(O_{2}\right)}$ is the diffusion coefficient of O_2_ in 0.1 M KOH (1.9 × 10^−5^ cm^2^ s^−1^), *ν* is the kinetic viscosity coefficient of electrolyte (0.01 cm^2^ s^−1^), $C_{\left(O_{2}\right)}$ is the bulk concentration of O_2_ in the electrolyte (1.2 × 10^−6^ mol cm^−3^), and *ω* is the electrode rotation speed (rpm).

The electron transfer number (*n*) and hydrogen peroxide yield (% H_2_O_2_) during the ORR can be determined by the RRDE technique:

$$H_{2}O_{2}\left(\%\right)=200\times \frac{\frac{I_{r}}{N}}{I_{d}+\frac{I_{r}}{N}}$$

$$n=4\times \frac{I_{d}}{I_{d}+\frac{I_{r}}{N}}$$

where *I_d_* is the disk current, *I_r_* is the ring current, and *N* = 0.37 is the current collection efficiency of the Pt ring.

### 3.2. Zn–Air Battery

The Zn–air battery was tested in a home-made electrochemical cell. The catalysts were loaded on a gas diffusion layer/carbon paper (2 mg cm^−2^). A Pt/C electrode with the same catalyst loading was also prepared as a comparative control. The commercial Zn foil was used as cathode and 6 M KOH was used as electrolyte containing 0.2 M Zn(Ac)_2_. All the measurements were performed on the as-constructed cell at room temperature with CHI 760D electrochemical workstation (CH Instruments, Shanghai, China).

The specific capacity is calculated using the data obtained from the measured constant current discharge curves.

$$specific\;capacity=\frac{current\;density\;\times \;test\;hours}{consumed\;zinc\;plate\;mass}$$

## 4. Results and Discussion

### 4.1. Synthesis Protocol

**Figure 1:**
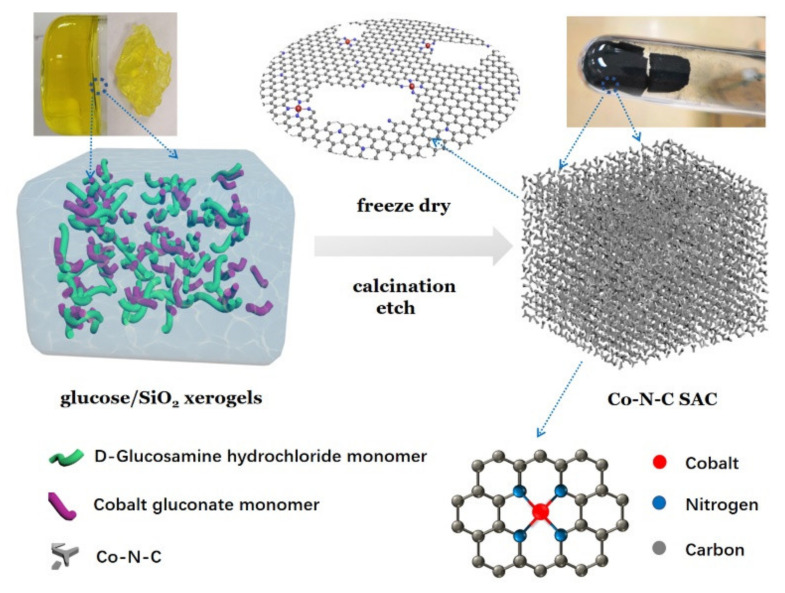
Schematic representation of the synthesis of Co-N-C SACs.

Figure 1 shows the schematic illustration of the overall synthetic process of Co-N-C SACs, which mainly includes three key steps. (*i*) First, there is the synthesis of the precursor in SiO_2_ so–gel. A classic Stöber approach was used to prepare the sol–gel, in which cobalt gluconate and glucosamine were mixed with TEOS (SiO_2_ precursor) for hydrolysis. This important step produces the SiO_2_ sol–gel with a uniform filling of Co, N, and C precursor solution in the 3D networks. (*ii*) Next, there is freeze drying, pyrolysis, and acid etching. Here, the volatiles (i.e., water, ethanol, and ammonia) adsorbed in the sol–gel system were readily evaporated upon freeze drying, leading to the formation of a SiO_2_ xerogel with cobalt gluconate and glucosamine molecules distributing homogeneously within the 3D frameworks of SiO_2_. Then, the xerogel system was pyrolyzed under an inert atmosphere to transfer the precursors into N-doped C and Co species supported by the SiO_2_ frames. In order to remove the SiO_2_ and undesirable substances, the annealed product was etched using hydrofluoric acid and treated by a post-thermal approach, eventually leading to the production of isolated single Co atoms anchored porous N-doped C materials. In contrast, a control N-C sample was synthesized using the same procedures without cobalt gluconate.

The 3D network of SiO_2_ xerogel acts as a hard template and protector during preparation, which can efficiently trap precursor molecules, stabilize the free migration of Co species, and prevent the congestion of Co nanoparticles/clusters [33,37,50,51]. The porosity and surface area of the resultant N-doped porous C networks can be greatly increased while removing the SiO_2_ xerogel and hence promote the exposure of active sites and mass transfer. Interestingly, the Co content can be facilely tuned-up to 2.38 wt% (confirmed by XPS). With this method, one is able to synthesize the Co-N-C SAC with a high amount of 100 g/batch at the laboratory scale.

### 4.2. Morphological Features

**Figure 2:**
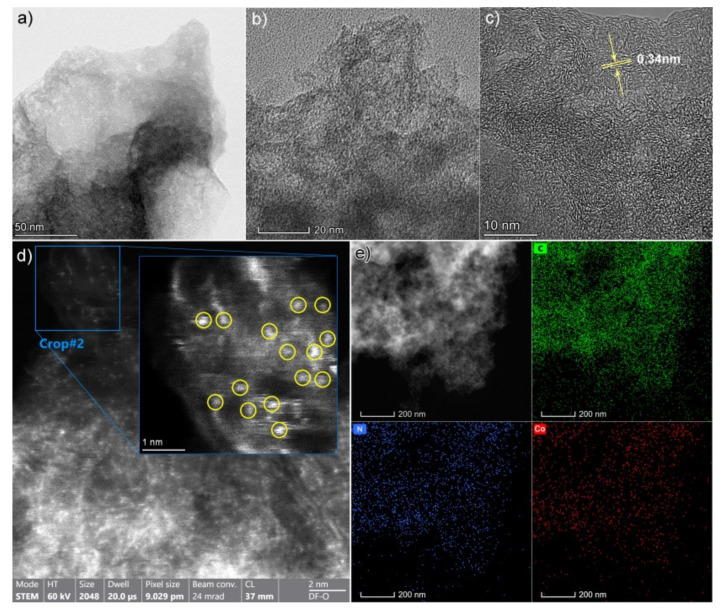
(**a**,**b**) TEM, (**c**) HRTEM, and (**d**) HAADF-STEM images of the Co-N-C SAC. Bright spots in (**d**) indicate the single Co metal atoms marked with a yellow circle dispersed in the carbon matrix. (**e**) Dark-field TEM image of the selected area for EDS elemental mapping images of C, N, and Co in the Co-N-C SAC.

The surface morphologies of Co-N-C SAC are presented in Figure 2a–e and Figure S1 via TEM and SEM. Co-N-C SAC shows the porous and disordered morphology (Figure 2a–c), and there are no obvious aggregates or nanoparticles. TEM and SEM images of the N-C sample also show the similar structure feature (Figure S2a,b). The high-resolution TEM image in Figure 2c reveals that the sample is constituted of distorted multilayer structures with an interplanar distance of ca. 0.34 nm, which is in good agreement with the typical features of graphite-like carbon (i.e., lattice spacing of {101} facet for graphite). To verify the formation of Co single atoms in the Co-N-C SAC, HAADF-STEM measurements were further performed. As exhibited in Figure 2d, plenty of bright spots with the size of around 0.2 nm are well distributed along the carbon support, corresponding to the individually dispersed Co atoms. This observation strongly confirms the presence of single atomic Co in the sample. Energy-dispersive spectrometer (EDS) mapping (Figure 2e) analysis was carried out, suggesting the Co, N, and C are distributed uniformly in the carbon matrix. Therefore, the TEM characterization of the Co-N-C SAC strongly indicates that our xerogel synthetic approach can be successfully applied to disperse Co single-atom species in the three-dimensional (3D) porous carbon networks.

### 4.3. Structural Features

**Figure 3:**
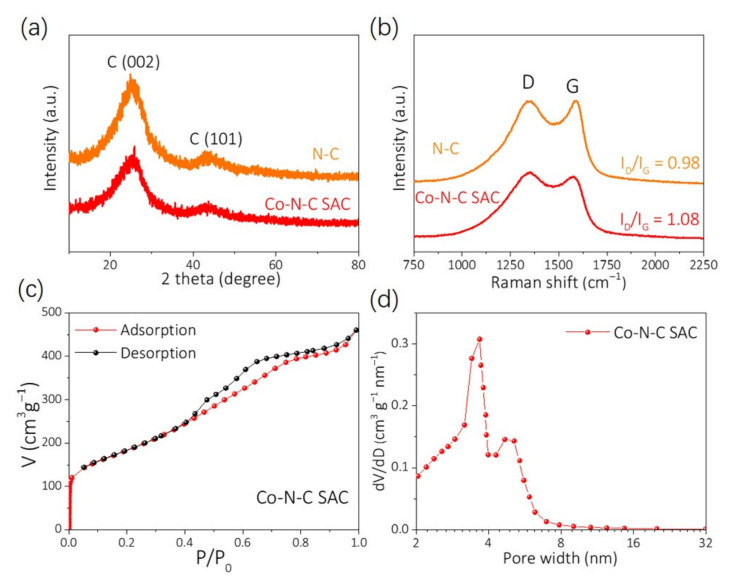
(**a**) XRD pattern and (**b**) Raman spectra of Co-N-C SAC and N-C. (**c**) Nitrogen adsorption/desorption isotherm and (**d**) BJH pore size distribution plot of Co-N-C SAC.

The samples were also examined using XRD spectroscopy. The XRD pattern (Figure 3a) of Co-N-C SAC and N-C shows wide peaks at ≈25.7° and ≈44.0°, which may be attributed to the diffractions of {002} and {101} planes of graphitic carbon, respectively. Obviously, there is no distinct diffraction peak induced by metallic Co or other Co-based crystals, hence confirming the formation of atomically dispersed Co single atoms in the samples. Complementary to TEM observations, the Raman spectra of the Co-N-C SAC and N-C offered the structural defect and chemical composition (Figure 3b), which closely link to their electrocatalytic performance of the ORR and OER. Figure 3b shows the deconvoluted spectra of the Co-N-C SAC and N-C sample, indicating a G band and D band at peaks of ≈1590 cm^−1^ and at ≈1350 cm^−1^, respectively. The calculated intensity ratios of I_D_/I_G_ for Co-N-C SAC and N-C are 1.08 and 0.98, respectively, which confirms the presence of amorphous carbon of Co-N-C SAC with a lower graphitization degree than the N-C one.

The porosity of the carbon support critically influences the mass transport between the catalyst surface and the bulk solution [42,43]. The N_2_ adsorption/desorption isotherm and type-IV adsorption isotherm for Co-N-C SAC are illustrated in Figure 3c. The Co-N-C SAC presented a steep increase in V_ads_ at a relatively low N_2_ pressure (P/P_0_ = 0–0.015) and a well-defined hysteresis loop at a higher N_2_ pressure (P/P_0_ = 0.4–0.9), indicating the coexistence of micro and mesopores [43,52]. The surface area of Co-N-C SAC is 737 m^2^ g^−1^ calculated from the isotherm via BET, and the Barret, Joyner, and Halenda (BJH) pore size distribution indicates that the pore diameters are in the range of 3–6 nm (Figure 3d). Figure S3 displays the cumulative pore volume. Obviously, Co-N-C SAC mainly contains micropores and mesopores with 2–10 nm size range, and the Co-N-C SAC shows a sharp increase in pore volume when the mesopores are less than 10 nm. The BET surface area analysis and TEM characterizations (Figure 2) suggest that the Co-N-C SAC consists of a homogeneous disordered 3D porous network architecture with a large amount of micropores and mesopores, in which the micropores host most of the active sites to drive the reaction [53,54], and mesopores mainly provide the channels for reactant exchange [43]. Therefore, the Co-N-C SAC is expected to show excellent catalytic performance in ZABs.

**Figure 4:**
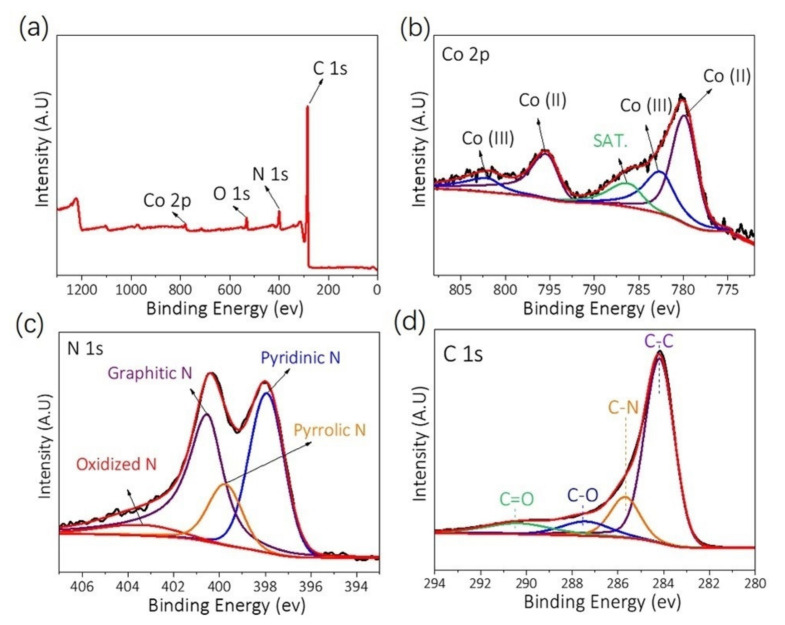
(**a**) XPS survey spectrum of Co-N-C SAC. Deconvoluted XPS spectra of (**b**) Co 2p, (**c**) N 1s, and (**d**) C 1s for Co-N-C SAC.

XPS measurements were carried out for studying the surface composition and chemical state of Co-N-C SAC. The XPS survey spectra of Co-N-C SAC show C 1s, N 1s, and Co 2p signals in the sample (Figure 4a). The high-resolution spectra of C 1s, N 1s, and Co 2p were also recorded for obtaining deep insight into the individual elements in Co-N-C SAC. As shown in Figure 4b–d, the Co 2p spectrum after deconvolution presents two pairs of peaks for Co^2+^ (795.6 and 780.1 eV) and Co^3+^ (802.1 and 782.7 eV) with a satellite peak at ≈786.3 eV, and no signal of metallic Co (Co^0^) is detected. The N 1s spectrum in Figure 4c can be deconvoluted into four distinct peaks, including the species of pyridinic-N (≈397.9 eV), pyrrolic-N (≈399.8 eV), graphitic-N (≈400.6 eV), and oxidized-N (≈403.3 eV). The N content in Co-N-C SAC was determined to be as high as 10.74 wt% (Table S1). It has been reported that the pyridinic-N plays a crucial role to generate Co-N_x_ active sites, while graphitic N affects the geometry and electronic structure of the carbon matrix [50,55,56]. The C 1s spectrum displays four peaks at 290.4, 287.5, 285.6, and 284.2 eV, which can be assigned to the C=O, C-O, C-N, and C-C group, respectively (Figure 4d).

### 4.4. Electrochemical Measurements

**Figure 5:**
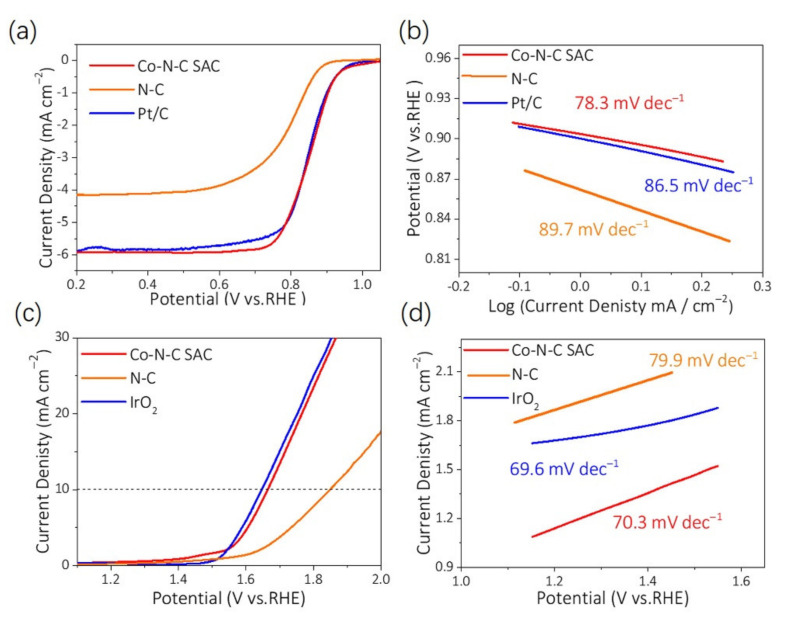
(**a**) ORR polarization curves and (**b**) corresponding Tafel plots of Co-N-C SAC. (**c**) OER polarization curves and (**d**) corresponding Tafel plots of Co-N-C SAC.

The liner sweep voltammograms (LSVs) and cycle voltammograms (CVs) of the synthesized samples were used to evaluate the ORR performance in N_2_- and O_2_-saturated KOH electrolyte. The Co-N-C SAC exhibits greatly improved performance of onset potential (E_onset_ = 1.01 V vs. RHE), as shown in Figure 5a, which are more active than a commercial 20% Pt/C (E_onset_ = 0.97 V vs. RHE), N-C (E_onset_ = 0.90 V vs. RHE) and the reference catalysts reported recently (Table S2). The Co-N-C SAC demonstrates the good ORR performance with the highest half-wave potential (E_1/2_) value of 0.851 V and an obviously enlarged diffusion-limited current density (J_lim_) about 5.92 mA cm^−2^ at 0.2 V vs. RHE (Figure 5a). The performance is similar to the 20% Pt/C catalyst (E_1/2_ 0.847 V, J_lim_ 5.89 mA cm^−2^) and better than N-C catalyst (E_1/2_ 0.794 V, J_lim_ 4.15 mA cm^−2^). Figure 5b shows the Tafel slopes of these samples, where Co-N-C SAC represents an attractive Tafel slope of 78.3 mV dec^−1^, which is smaller than that of Pt/C (86.5 mV dec^−1^) and N-C (89.7 mV dec^−1^), indicating that Co-N-C SAC shows more favorable ORR kinetics ascribed to the acceleration of mass and electron transport in 3D interconnected mesopores carbon networks of Co-N-C SAC [53,54]. Furthermore, the calculated electron transfer number (n) (Figure S4) was obtained according to Koutecky–Levich (*K*–*L*) plots for studying the ORR kinetics of Co-N-C SAC. The n is calculated to be close to 4, indicating that the catalyst undergoes a catalytic process via a four-electron pathway with high-efficiency. Figure S5 shows that the H_2_O_2_ yield is lower than 10% detected on the Pt ring.

To evaluate the bifunctionality of the catalysts for rechargeable ZAB, the OER performances of the catalysts were also measured in a 0.1 M KOH electrolyte. As illustrated by the LSV results in Figure 5c,d, the overpotentials required to reach the current density of 10 mA cm^−2^ is 430 mV, 618 mV, and 400 mV for Co-N-C SAC, N-C, and IrO_2_, respectively. The overpotential of Co-N-C SAC is lower than the N-C sample and comparable to that of IrO_2_. Additionally, Co-N-C SAC demonstrates a lower Tafel slope of 70.3 mV dec^−1^, which is significantly smaller than those of N-C (79.9 mV dec^−1^), suggesting a faster OER catalytic kinetics (Figure 5d) perhaps on the basis of the higher amount of atomically dispersed Co species and larger surface area.

### 4.5. Zn–Air Battery Performance

**Figure 6:**
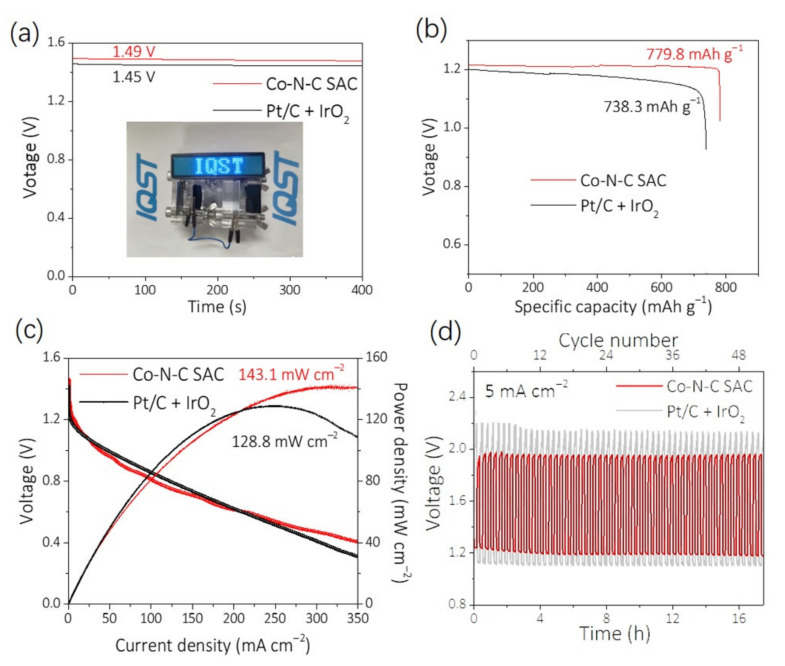
(**a**) Open-circuit plots of Co-N-C SAC and 20% Pt/C + IrO_2_ (inset: Optical image of an LED light array powered by two ZABs in series using Co-N-C SAC as the air cathode). (**b**) Specific capacities of the ZABs using Co-N-C SAC and 20% Pt/C + IrO_2_ as catalysts, which are normalized to the mass of the completely consumed Zn. (**c**) Polarization and power density curves for primary ZABs. (**d**) Charge–discharge cycling performance of Co-N-C SAC-based ZABs and 20% Pt/C+IrO_2_-based ZABs with a duration of 20 min per cycle at 5 mA cm^−2^.

Beyond traditional electrochemical investigations in standard three-electrode systems, the homemade primary and rechargeable ZABs were also fabricated to further examine the performance of Co-N-C SAC as air cathode catalyst. The ZAB was equipped with 6 M KOH containing 0.2 M Zn(Ac)_2_ as electrolyte, and a zinc plate and Co-N-C SAC-loaded carbon paper (1 mg/cm^2^) was regarded as the anode and air cathode, respectively. In contrast, the 20% Pt/C + IrO_2_ counterpart was also assembled. The open-circuit voltage of the Co-N-C SAC-based battery is 1.49 V, which is superior to the Pt/C + IrO_2_ counterpart of 1.45 V (Figure 6a). Furthermore, two homemade ZABs light up an LED (1.5–5 V) panel lamp, demonstrating the successful operation of the ZAB (Figure 6a inset). The specific capacity of the ZAB using a cathodic Co-N-C SAC-based catalyst (Figure 6b) is calculated to be 779.8 mA h g^−1^ at 10 mA cm^−2^, which is slightly higher than the system using 20% Pt/C + IrO_2_ with 738.3 mA h g^−1^. The corresponding energy density of the Co-N-C SAC-based battery reaches 942 Wh kg^−1^ at 10 mA cm^−2^, which is also superior to that of the Pt/C-based one (884 Wh kg^−1^).

The power density of battery is another vital parameter, reflecting the quality of the battery. Figure 6c presents the discharging polarization curves of the Co-N-C SAC and 20% Pt/C + IrO_2_-based ZAB; the maximum power density of the Co-N-C SAC-based battery reaches 143.1 mW cm^−2^, exceeding that of its 20% Pt/C + IrO_2_-based counterpart (128.8 mW cm^−2^) and most of the reported non-precious metal catalysts in this field [49]. The stability of the Co-N-C SAC-based battery was also assessed by a rechargeable ZAB, as shown in Figure 6d, where the initial discharging voltage is 1.18 V and charging voltage is 1.96 V for the Co-N-C SAC-based battery at the current density of 5 mA cm^−2^. The charging–discharging voltage gap of the Co-N-C SAC-based battery maintains the initial overpotential of 0.78 V without any decay after a consecutive 54 cycles’ scanning, indicating the long-term stability of the Co-N-C SAC-based ZAB. Obviously, the Co-N-C SAC-based battery delivers a lower charge–discharge voltage gap compared with the 20% Pt/C + IrO_2_-based one (1.01 V), indicating a better rechargeability. All these outcomes suggest that the Co-N-C SAC can be employed as a promising electrode material for the potential application in the rechargeable ZABs.

## 5. Conclusions

In summary, we have presented a facile silica xerogel-assisted synthetic strategy to disperse Co single atoms on a N-doped porous network carbon as electrocatalyst for high-efficiency ORR/OER in alkaline medium. The synthesized Co-N-C SAC demonstrates excellent bifunctional performance due to the high-content Co-N_x_ active sites, massive mesopores, and micropores of carbon matrix. The assembled Zn-air battery using the Co-N-C SAC exhibits outstanding specific energy of 779.8 mA h g^−1^ at 10 mA cm^−2^, excellent power density of 143.1 mW cm^−2^, and moderately durability. This robust silica xerogel strategy is expected to fabricate various SACs with dense accessible active sites, high porosity, and surface area for broad application.

## Data Availability

The data that support the findings of this study are available from the corresponding authors upon reasonable request.

## Supplementary Materials

The following supporting information can be downloaded at: https://www.mdpi.com/article/10.3390/nano12030381/s1, Figure S1: (a,b) SEM images of the Co-N-C SAC. Figure S2: (a) SEM and (b) TEM images of the N-C sample. Figure S3: Cumulative pore volume for Co-N-C SAC. Figure S4: (a) ORR LSV at different rates, (b) *K*–*L* plots of Co-N-C SAC. Figure S5: The H_2_O_2_ yield and number of electron transfer at Co-N-C SAC and Pt/C. Table S1: Mass fraction content of C, N, O, and Co in Co-N-C SAC extracted from XPS measurements. Table S2: The onset potential of each catalyst in this work during ORR compared to the recent literature.
