# Supplementary material for: Bifunctional Single-Atom Cobalt Electrocatalysts with Dense Active Sites Prepared via a Silica Xerogel Strategy for Rechargeable Zinc–Air Batteries
Source: Nanomaterials (Basel). 2022 Jan 24;12(3):381. doi: 10.3390/nano12030381 (PMC8840332; doi:10.3390/nano12030381)
Supplement: Supplementary file 1 [file nanomaterials-12-00381-s001.zip › nanomaterials-1534951-supplementary.pdf]

nanomaterials

MDPI

Supplementary Materials

Bifunctional Single-atom Cobalt Electrocatalysts with Dense Active Sites Prepared via a Silica Xerogel Strategy for Rechargeable Zinc-Air Batteries

Lijuan Wang [1], Zixiang Xu [1], Tingyu Peng [2], Maosong Liu [1,*], Long Zhang [1,*] and Jianming Zhang [1,*]

[1] Institute of Quantum and Sustainable Technology (IQST), School of Chemistry and Chemical Engineering, Jiangsu University, Zhenjiang, 212013, China; 2211912020@stmail.ujs.edu.cn (L.W.); 3191303039@stmail.ujs.edu.cn (Z.X.)
[2] Instrumental Analysis Center, Jiangsu University, Zhenjiang, 212013, China; 1000005654@ujs.edu.cn
* Correspondence: 2112012005@stmail.ujs.edu.cn (M.L.); longzhang@ujs.edu.cn (L.Z.); zhangjm@ujs.edu.cn (J.Z.)

Keywords: single-atom catalysts; ORR/OER; electrocatalysis; zinc-air battery; xerogel

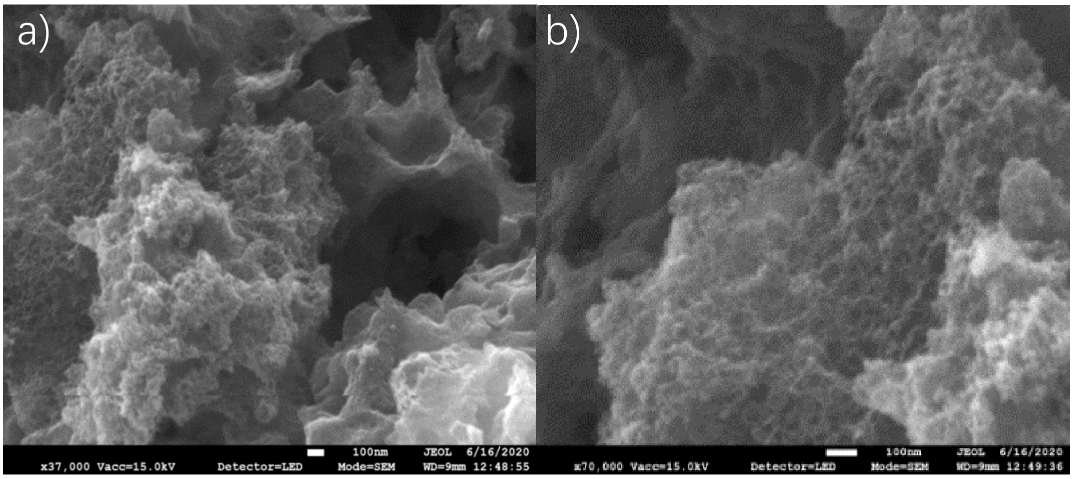

Figure S1. (**a**,**b**) SEM images of the Co-N-C SAC.

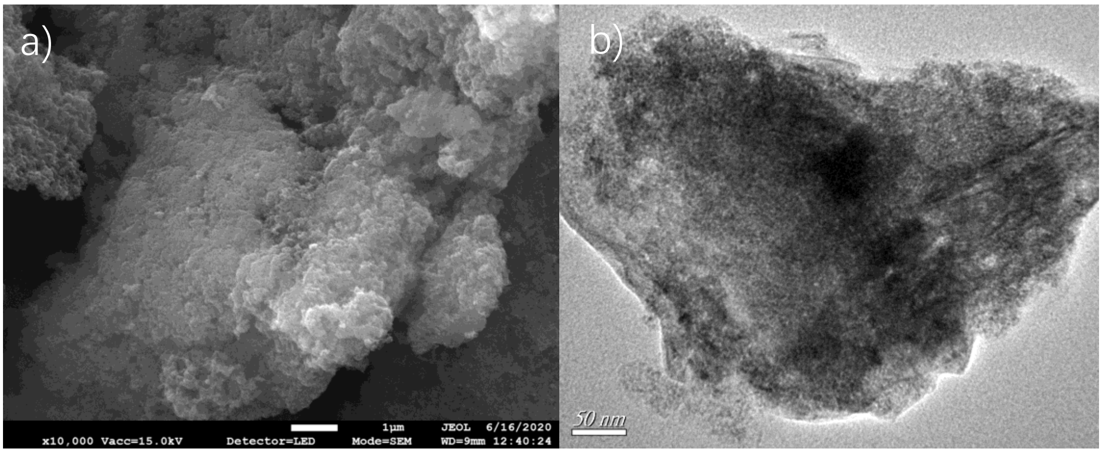

Figure S2. (**a**) SEM and (**b**) TEM images of the N-C sample.

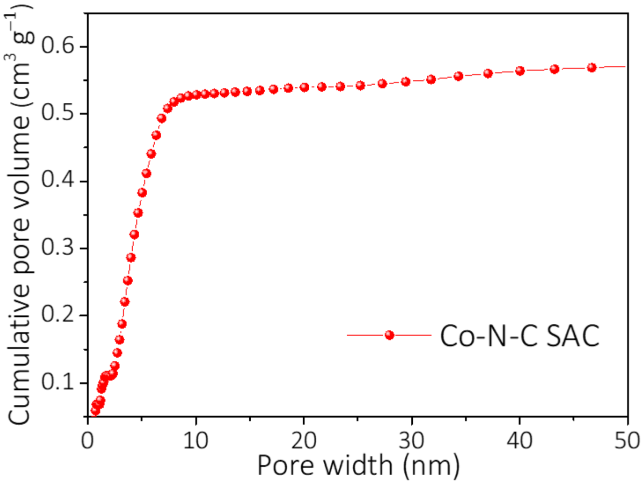

Figure S3. Cumulative pore volume for Co-N-C SAC.

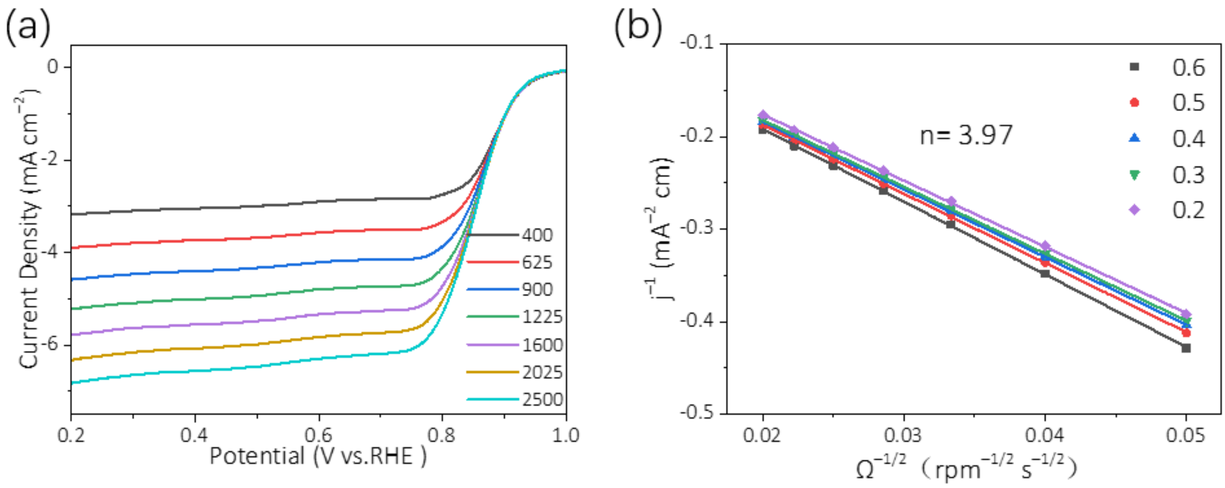

Figure S4. (a) ORR LSV at different rates, (**b**) K-L plots of Co-N-C SAC.

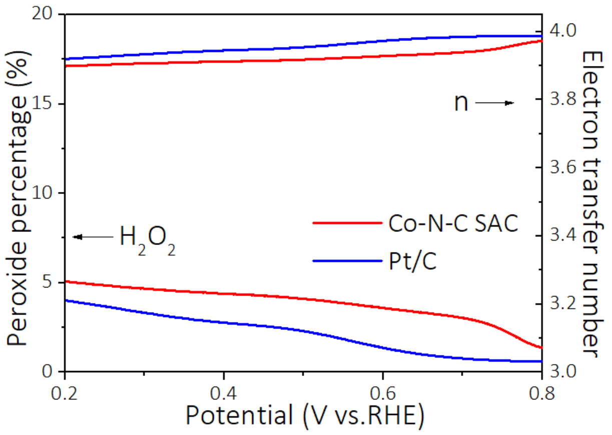

Figure S5. The H_2O_2 yield and number of electron transfer at Co-N-C SAC and Pt/C.

Table S1. Mass fraction content of C, N, O and Co in Co-N-C SAC extracted from XPS measurements.

Catalysts	C	N	O	Co
Co-N-C SAC	81.62 wt%	10.74 wt%	3.03 wt%	2.38 wt%

Table S2. The onset potential of each catalyst in this work during ORR compared to the recent literature.

Entry	Catalyst	E_{onset} (V *vs.* RHE)	Reference
1	Co-N-C SAC	1.01	This work
2	N-C	0.90	
3	Pt/C	0.97	
4	Co-N-OCC	0.87	[1]
5	act-Co/N/C	0.943	[2]
6	Co/VN@NC	0.954	[3]
7	Fe-NC	0.963	[4]
8	$Fe_3C/Co(Fe)O_x$@NCNT	0.97	[5]
9	Co-NHCS	0.99	[6]
10	BCNT/Co-800	1.12	[7]

References

1. Xiang, Y.; Yang, T.; Tong, K.; Fu, T.; Tang, Y.; Liu, F.; Xiong, Z.; Si, Y.; Guo, C. Constructing flexible and self-standing electrocatalyst for oxygen reduction reaction by in situ doping nitrogen atoms into carbon cloth. *Appl. Surf. Sci.* **2020**, *523*, 146424.
2. Zhu, Y.; Miyake, K.; Shu, Y.; Gabe, A.; Hirota, Y.; Uchida, Y.; Tanaka, S.; Morallón, E.; Cazorla-Amorós, D.; Nishiyama, N. Anchoring a Co/2-methylimidazole complex on ion-exchange resin and its transformation to Co/N-doped carbon as an electrocatalyst for the ORR. *Catal. Sci. Technol.* **2019**, *9*, 578–582.
3. Cen, T.; Qiu, L.; Ye, Z.; Peng, X.; Liu, Y.; Yuan, D. Co/VN heterostructure coated with holey interconnected carbon frameworks as bifunctional catalysts. *Int. J. Hydrogen Energ.* **2021**, *46*, 3337–3345.
4. Wang, Y.; Pan, Y.; Zhu, L.; Yu, H.; Duan, B.; Wang, R.; Zhang, Z.; Qiu, S. Solvent-free assembly of Co/Fe-containing MOFs derived N-doped mesoporous carbon nanosheets for ORR and HER. *Carbon* **2019**, *146*, 671–679.
5. Wang, M.; Qian, T.; Liu, S.; Zhou, J.; Yan, C. Unprecedented Activity of Bifunctional Electrocatalyst for High Power Density Aqueous Zinc–Air Batteries. *ACS Appl. Mater. Interfaces.* **2017**, *9*, 21216–21224.
6. Chen, S.; Cheng, J.; Ma, L.; Zhou, S.; Xu, X.; Zhi, C.; Zhang, W.; Zhi, L.; Zapien, J.A. Light-weight 3D Co–N-doped hollow carbon spheres as efficient electrocatalysts for rechargeable zinc–air batteries. *Nanoscale.* **2018**, *10*, 10412–10419.
7. Liu, X.; Yang, W.; Chen, L.; Liu, Z.; Long, L.; Wang, S.; Liu, C.; Dong, S.; Jia, J. Graphitic Carbon Nitride (g-C_3N_4)-Derived Bamboo-Like Carbon Nanotubes/Co Nanoparticles Hybrids for Highly Efficient Electrocatalytic Oxygen Reduction. *ACS Appl. Mater. Interfaces.* **2020**, *12*, 4463–4472.
